# Assessment of the Dietary Intake Changes in Patients with Head and Neck Cancer Treated with Radical Radiotherapy

**DOI:** 10.3390/nu16132093

**Published:** 2024-06-30

**Authors:** Agnieszka Surwiłło-Snarska, Aleksandra Kapała, Dorota Szostak-Węgierek

**Affiliations:** 1 Department of Clinical Nutrition, Maria Sklodowska-Curie National Research Institute of Oncology, 02-781 Warsaw, Poland; aleksandra.kapala@nio.gov.pl; 2 Department of Oncology Diagnostics, Cardio-Oncology and Palliative Medicine, Maria Sklodowska-Curie National Research Institute of Oncology, 02-781 Warsaw, Poland; 3 Department of Clinical Dietetics, Faculty of Health Sciences, Medical University of Warsaw, E Ciołka 27, 01-445 Warsaw, Poland; dorota.szostak-wegierek@wum.edu.pl

**Keywords:** head and neck cancers, radical radiotherapy, nutritional assessment, malnutrition, clinical nutrition, dietary counseling

## Abstract

Background: Patients during radiotherapy due to head and neck cancers experience a lot of side effects which may have a considerable impact on the patients’ ability to meet individual daily energy demands by means of oral diet. Methods: The study included 104 head and neck cancer patients who qualified for radical radiotherapy. Radical treatment takes 6 weeks and every week the patients were assessed for dietary intake. The subjects were covered with the constant care of a dietician, received FSMP (food for special medical purposes), and, if necessary, enteral nutrition. Results: In the first week of treatment, the patients, from the kitchen diet alone, met 91.5% of the energy demand, while in the last week of treatment, only 40.9%. After introducing the FSMP or enteral nutrition, the patients met 120% of the demand in the first week of therapy and 95% in the last week, respectively. The patients who followed the dietary recommendations were characterized by significantly lower weight loss (3.07 kg) compared to non-adherent patients (5.56 kg). Conclusions: The used therapy significantly contributed to decreasing nutritional intake in the subsequent weeks of treatment. On the other hand, incorporating FSMP in the diet and enteral nutrition with industrial diets significantly increased the fulfilled energy demand of patients.

## 1. Introduction

The term “tumors of the head and neck area” refers to a group of cancers originating in the following organs: mouth, lip, pharynx (oral, laryngeal, and nasal parts), nasal sinuses, nasal cavities, salivary glands, and ear. The most common histological type of head and neck organ tumors is squamous cell carcinoma, which develops from mucosal epithelium [1,2].

Head and neck cancers account for 5–6% of all the malignant tumors in Poland. According to the National Cancer Registry Report, they affect men more often than women, with an increase in incidence in people over 50. The exception is nasopharyngeal squamous cell carcinoma, where the highest incidence is in young people (20–35 years of age). The most common cancer of the head and neck is the cancer of the larynx [3].

Risk factors for developing head and neck cancer are well known. These include cigarette smoking, both active and passive, and alcohol consumption. These are responsible for 70% of the incidence. Other risk factors include a lack of proper oral hygiene, mechanical irritation of the oral mucosa (e.g., through ill-fitting dentures), and viral infections, mainly human papilloma virus (HPV) and Epstein–Barr virus (EBV). Dietary factors that increase the risk of developing head and neck cancer include low consumption of fruits and vegetables [4,5].

Radical treatment options for patients diagnosed with head and neck cancer include surgery or radiation therapy. These methods are also used as combined treatment with radiotherapy complementing surgery. Additionally, in patients with more advanced disease (locally and/or regionally), radiotherapy is used in combination with chemotherapy. Radical radiotherapy (alone or in combination with chemotherapy) is a long-term treatment, usually carried out in 28–30 fractions, so it usually lasts 6 to 7 weeks. Radical radiotherapy (ionizing) is carried out using 3D conformal irradiation technology, with a preference for IMRT (intensity-modulated beam irradiation). The treatment of head and neck cancer is fraught with high toxicity; therefore, nutritional intervention is an integral part of it [1,2].

Ionizing radiation used in diagnosis and cancer radiotherapy is well known and has both beneficial and harmful effects on biological systems. The method by which radiation interacts with a biological system may be direct or indirect. Damage can occur due to the direct ionization of the DNA molecule itself or indirectly through the formation of toxic products, such as free radicals, hydrogen peroxide, hydroperoxy radicals, and hydroperoxy ions, that diffuse from the site of formation and interact with any molecules in their path. The risk of radiation damage to normal tissues increases with the number of radiotherapy fractions, the total dose, and the volume of tissues that have been irradiated. The main challenge is to select the appropriate maximum doses of radiation reaching the cancer cells while minimizing the damage to healthy tissue [6,7].

The appearance of radiation reaction in the mucous membranes and skin in the irradiated area translates into the development of swallowing disorders ranging from dysphagia and odynophagia to aphagia. A consequence of salivary gland damage is the development of xerostomia. In addition, patients experience anorexia, nausea and vomiting; trismus; and an increased risk of aspiration during treatment. As early as the 3rd week of treatment, the discomfort worsens, and patients report three or more problems with their ability to eat efficiently, which directly translates into weight loss [8,9].

The problems described above often prevent patients from receiving effective oral nutrition, and it is necessary to incorporate enteral feeding via artificial access (a percutaneous endoscopic gastrostomy created before treatment or a nasogastric tube inserted during therapy). Patients require the care of a nutritionist, speech therapist, physical therapist, psychologist, nurse, and social worker during treatment. The goal of multispecialty teams is not only to improve treatment outcomes, but also to improve the patient’s quality of life [10].

The main purpose of this study was to evaluate in detail the intake of energy, protein, and other macro- and micronutrients during each successive week of radiation therapy. To the authors’ best knowledge, these are data that are limited in the literature.

## 2. Materials and Methods

The study was conducted between 2018 and 2019. It was designed as an observational clinical trial involving 104 adult patients diagnosed with primary head and neck organ cancer who were qualified for radical treatment: radiotherapy alone or radiochemotherapy or radiotherapy with cetuximab. Consecutive hospitalized patients treated at the Head and Neck Cancer Department at the National Research Institute of Oncology in Warsaw were included in the study. The study received a positive opinion from the Bioethics Committee (No. 4/2018) at the National Research Institute of Oncology in Warsaw.

### 2.1. Inclusion Criteria

Inclusion criteria for the study included diagnosed cancer of the organs of the head and neck qualified for treatment with radical radiation therapy, hospitalization for the duration of the entire treatment, and patient consent to participate in the study.

Exclusion criteria for the study included palliative treatment, prior radiation therapy in the head and neck region, other concurrent malignant neoplasm, outpatient treatment, and the lack of patient consent to participate in the study.

### 2.2. Scheme of Implementation of the Survey

The study lasted 6 weeks and was carried out using a prospective observational method with the tools detailed below. Throughout the period of radical treatment, the patients underwent weekly dietary assessments. In addition, each patient had an individual dietary consultation.

### 2.3. Dietary Assessment

The assessment of dietary intake and intake of macro- and micronutrients was carried out using the 24 h dietary interview method. During the dietary consultation, the patient was asked what products, foods, and drinks and in what quantities (in home measures) he/she had consumed the previous day. The Food and Nutrition Institute’s “Photo Album of Products and Foods” was used to assess portion sizes [11].

Information was also collected on the oral use of Food for Special Medical Purposes (FSMP) and the amount and type of enteral nutrition (if the patient was receiving nutrition in this way); in addition, an intravenous glucose supply was included.

The 24 h dietary interview method is a retrospective, questionnaire-based, qualitative–quantitative method that provides accurate information on the amount and type of food and beverages consumed. The method determines current consumption [12]. The data obtained were entered into the DIETA 6.0 computer program developed by the Institute of Food and Nutrition in Poland, which made it possible to estimate the intake of energy and the main nutrients, i.e., protein, fat, and carbohydrates, as well as selected vitamins and minerals. The values obtained were reduced by technological and plate losses.

The analysis also identified the patients who were not consuming the recommended amount of energy, which was the basis for implementing an appropriate dietary intervention to increase energy supply through the use of fortified foods, oral application of FSMP, or enteral nutrition (EN). It was assumed that the daily energy requirement for normal-weight and malnourished patients was 30 kcal/kg/d of current body weight, while for overweight and obese patients, the caloric requirement was converted to due body weight [10,13].

### 2.4. Dietary Consultation

The patients included in the study received regular care from a dietitian throughout the treatment period. The first dietary consultation was held on the first day of radical radiotherapy, subsequent consultations were held at the beginning of the second, third, fourth, and fifth weeks of treatment, and the sixth consultation was held on the last day of treatment. If nutritional problems arose, the patients could receive an additional dietary consultation at any time during treatment. The dietitian’s consultation consisted of a detailed history in accordance with the established practice, the estimation of individual energy requirements, and the selection of a dietary supply route depending on the patient’s current clinical condition. On the day of the start of radiation therapy, each patient received in writing detailed guidelines for the selection of foods and the principles of diet composition.

From the first day of radiation therapy, each patient, regardless of the baseline nutritional status, received special medical nutrition (2 packs per day of a high-protein or high-energy diet, which translated into providing 600–800 kcal/d and 36 protein/d in addition to the baseline diet). In the case of pre-treatment weight loss greater than 10% of the baseline body weight, the patients additionally received a fat supplement providing 400 kcal/d.

With regular medical and dietary supervision, the patients whose oral food intake fell below 60% of the energy requirements were ordered enteral nutrition via PEG or nasogastric tube insertion. The diets routinely used for enteral nutrition are high-energy or high-protein industrial diets. In the case of special clinical situations, such as diabetes mellitus, liver failure, and intolerance to polymeric diets, there was the possibility of individual selection of the diet composition to suit the patient’s current condition.

The patients included in the study also benefited from nursing care (this was especially true of care for access to nutrition, and the delivery of oral and enteral formula), and had the opportunity to receive care from a speech therapist, physiotherapist, and psychologist.

For the purposes of the study, medical data were additionally collected from the patient’s medical history regarding comorbidities, medications used, the length of hospitalization, and the complications of treatment. A history was taken, based on which the presence of gastrointestinal symptoms accompanying the underlying disease and its treatment (including nausea, vomiting, constipation, and diarrhea) was noted.

### 2.5. Statistical Analysis

The mean (M), standard deviation (SD), median (Me), and minimum (min.) and maximum (max.) values were used for descriptive statistics. The Shapiro–Wilk test was used to assess the normality of the data distribution. To assess the statistical significance of changes, the Student’s *t*-test was used for the variables whose distribution approximated a normal distribution, while the Wilcoxon test was used for the variables whose distribution deviated from a normal distribution. If the assumptions allowing the use of parametric tests were not met (a lack of normality of the distributions of the variables confirmed by the Shapiro–Wilk test, or a lack of sphericity of the variables confirmed by the Mauchly test), comparisons were made using Friedman’s non-parametric ANOVA test. To determine the strength and direction of the relationship between the parameters analyzed in the study, Pearson’s linear correlation coefficient was used (in the case of conformity to a normal distribution), in the case of non-normality of the distribution, the rho-Spearman correlation coefficient was tested. The significance level was taken as α < 0.05. The statistical analyses were performed using the Jamovi software version 1.2.22.

## 3. Results

### 3.1. Characteristics of the Study Group

The table below (Table 1) presents the characteristics of the study group.

The mean age of the patients was 58.5 ± 10.8 years. The mean weight of the patients at the beginning of treatment was 74.6 ± 14.0, and BMI was 25.65 ± 4.34 kg/m^2^.

### 3.2. Effects of Treatment on Energy and Macro- and Micronutrient Dietary Intake

Data on the average daily intake of energy, macronutrients, and micronutrients in the following weeks of treatment, excluding FSMP and enteral nutrition, are shown in the table (Table 2).

**Figure 1:**
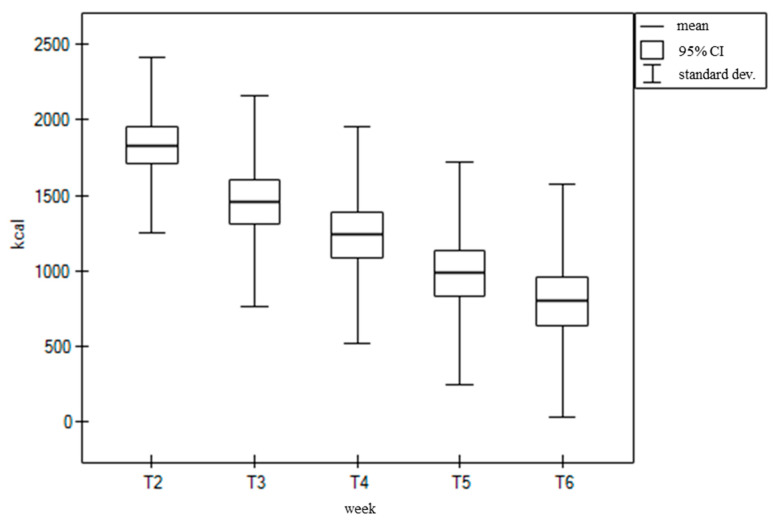
Daily dietary energy value (kcal) for consecutive weeks of treatment (from T2 to T6).

The average daily value of energy and nutrient intake in the following weeks of the patients’ treatment was significantly reduced in the vast majority of cases. The average value of energy consumed after the first week of the patients’ treatment was 1847.01 kcal per day. By the final week, this average had dropped to just 809.47 kcal per day, which was 43.8% of the value from T2. The changes that occurred in the overall energy intake during the following weeks of treatment are shown in the following figure (Figure 1).

Data on energy, macronutrient, and micronutrient intake for each week of treatment, together with FSMP and enteral nutrition, are shown in the table below (Table 3).

**Figure 2:**
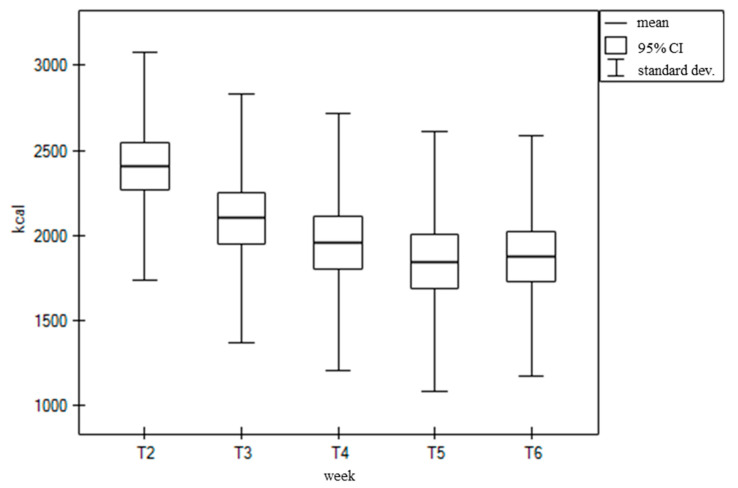
Daily dietary energy value (kcal), together with FSMP and EN, in consecutive weeks of treatment (from T2 to T6).

The intake of energy and macro- and micronutrients in the diet, including FSMP and enteral nutrition, shows a much smaller range of decreases than when they were consumed with the kitchen diet alone. In many cases, the intake levels were higher at T6 compared to T2. This includes calcium (132.8%), iron (116.3%), vitamin E (155.7%), and vitamin C (107.1%). The daily energy value of the diet, however, dropped from an average of 2432.2 kcal at T2 to 1889.97 kcal at T6. Changes in the energy intake during the following weeks of treatment are shown in the figure (Figure 2).

### 3.3. Energy Consumption vs. Individual Energy Needs

Data on the energy consumed by the patients, compared to the average daily energy requirements, are shown in the following table (Table 4).

**Figure 3:**
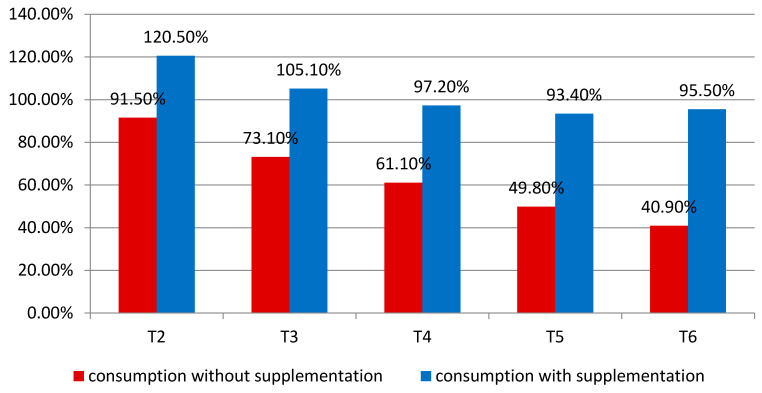
Energy intake with and without FSMP and EN in consecutive weeks of treatment, as a percentage of daily energy requirements.

There was a significant decrease in energy intake during the subsequent weeks of the patients’ treatment. At T2, the average intake without FSMP and EN accounted for 91.5% of the patients’ average requirements, while at T6, this ratio dropped to only 40.9% of the requirements. For intake with FSMP and EN, in the second week, the average energy value of the meals consumed was 120.5% of the requirement. In the last week of treatment, intake from FSMP and EN accounted for 95.5% of the energy requirements. These data are presented in the figure (Figure 3).

The therapy used significantly contributes to the reduction in oral food intake in the following weeks of treatment. In the last week of treatment, the patients realized only 40.9% of their energy requirements. In contrast, the inclusion of FSMP and enteral nutrition in the diet significantly increased the degree of the realization of the patients’ energy requirements.

### 3.4. The Stage of Treatment at Which the Inability to Maintain Daily Food Intake by Oral Route above 60% of Daily Energy Requirements Appears, Which Is an Indication for the Inclusion of Enteral Nutrition by Artificial Route (Tube or PEG)

The table shows the percentage of the subjects consuming by oral route more than 60% of their daily energy requirements in the following weeks of treatment (Table 5).

**Figure 4:**
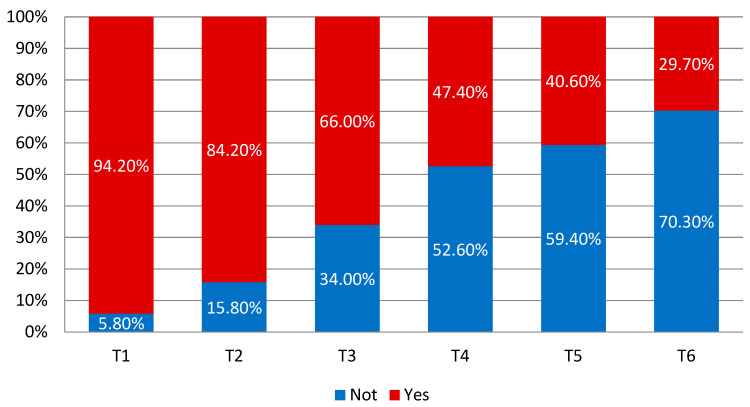
Realization of oral intake of 60% of energy requirements by patients.

The proportion of the patients consuming orally more than 60% of their daily energy requirements declined steadily in the subsequent weeks of the therapy administered. In the first week, 94.2% of the patients met this criterion, while in the last week (T6) only 29.7% of the patients did. The weighted average of the time elapsed before the onset of the inability to consume 60% of the daily energy requirements was 4.5 weeks. Differences in the percentage of the patients realizing an energy intake above 60% of the requirements in successive weeks of treatment proved to be statistically significant (<0.001). The changes occurring in this regard are shown in the figure (Figure 4).

### 3.5. Degree of Patient Adherence to Dietary Recommendations

The table shows the data on the daily energy value of the recommended food for special medical purposes (FSMP) and enteral nutrition (EN); the daily energy value of the FSMP and EN preparations actually taken, and the difference between these values in the subsequent weeks of treatment (Table 6).

During the following weeks of the patients’ treatment, the average daily energy value of the prescribed FSMP and EN preparations increased. At the beginning of treatment, it was 749.7 kcal (SD = 332.7), while in the last week, it was already 1340.4 kcal (SD = 572.82). The energy values of FSMP and EN taken by the patients in the following weeks also gradually increased. In the second week, the patients consumed an average of 595.8 kcal per day (SD = 420.34) in this form, while in the last week, they consumed 1096.8 kcal (SD = 579.47). The increase between T2 and T6 was thus 84.1%.

During the following weeks of treatment, there was a trend toward an increase in the difference between the energy value of the recommended FSMP and EN preparations and that actually taken. At T2, the average difference was 153.0 kcal (SD = 291.59), while at T6 it was already 243.7 kcal (SD = 465.60). This represents an increase between T2 and T6 of 59.3%. Differences between the daily energy values of the recommended and actual FSMP and EN intake in the successive weeks of treatment proved to be statistically significant (*p* < 0.001).

### 3.6. Reasons for Non-Compliance with FSMP Application Recommendations

The patients gave up taking the recommended food for special medical purposes mainly due to a feeling of fullness (35.7%), nausea (33.3%), and a lack of taste acceptance (28.6%). Less frequently, the reason for non-adherence was diarrhea (2.4%).

### 3.7. Degree of Implementation of Recommendations

Those completing at least 75% of the recommended dose of FSMP or enteral nutrition were counted as adhering to dietary recommendations. Sixty-one percent of the patients adhered to the dietary recommendations. Below the established threshold were 38.5% of the study participants.

Data on weight loss between T1 and T6, depending on adherence to dietary recommendations, are shown in the following table (Table 7).

Those who followed the dietary recommendations lost an average of 3.07 kg (SD = 2.32) during the treatment period, while those who did not follow the recommendations lost 5.56 kg (SD = 3.40). The average compliance rate with dietary recommendations was 78.0% (SD = 28.6%).

## 4. Discussion

When analyzing the clinical situation of patients with head and neck cancer, it is impossible to ignore the impact of the acute and late side effects of radical radiotherapy, which include oral mucositis, xerostomia, dysphagia, odynophagia, taste and smell disorders, a loss of appetite, and nausea. The development of the aforementioned ailments directly translates into a worsening of an already mostly abnormal nutritional state [14,15,16,17].

One of the determinants of nutritional status is nutrition, both quantitative and qualitative. The ability for efficient oral nutrition in patients with head and neck cancer is mainly determined by the location and stage of the disease and the stage of radical treatment (the severity of dysphagia and oral mucositis play an important role). Due to the problems described, food intake is often reduced, leading to weight loss. It should be noted that the side effects of radiation therapy worsen with each successive week of treatment despite the use of modern drugs designed to alleviate them. They adversely affect the quantity and quality of food consumed and nutritional status. Therefore, it is extremely important to match dietary advice with the current complaints reported by the patient [17,18].

It is estimated that an energy intake of less than 25 kcal/kg/day is associated with a high risk of malnutrition. Nutrition assessment should be conducted regularly so that the optimal dietary intake can be determined and adapted to the current clinical situation [19,20]. According to the “NutritionDay” multicenter study conducted by means of a one-day nutritional assessment in more than 300 European hospitals, hospital meals do not cover the energy needs of hospitalized patients. The authors of the study report that energy intake in 43% of the subjects did not exceed 1500 kcal per day [21].

Given the poor quality of hospital meals, patients’ aversion to food served in hospitals, and the undeniable impact of radical radiotherapy on the ability to eat orally, our study analyzed data on oral food intake. During the first week of radiotherapy, considering only oral diet, the patients took in an average of 1847 kcal and 70.3 g of protein per day. With each subsequent week of radiotherapy, energy and protein intake successively decreased shaping up in the last week of treatment at only 809.5 kcal and 36.5 g of protein per day. The percentage of the realization of energy requirements during the treatment decreased from 91.5% to 40.9%. The changes described above were statistically significant. A similar relationship was observed by van Berg et al. [22]. The energy intake of the patients treated with radiochemotherapy on the day of the completion of therapy was lower by 1234 kcal compared to baseline, which translated into a supply of only 19 kcal/kg/d.

In our study, the supply of other nutrients also successively decreased with the duration of radiotherapy. A decrease in the average intake of total fat, long-chain polyunsaturated fatty acids, carbohydrates, sodium, iron, zinc, copper, vitamin A, vitamin E, thiamin, niacin, vitamin B6, vitamin C, and vitamin D was observed. During the last week of radiotherapy, the average intake of energy; protein; carbohydrates; total fat; vitamins A, E, C, D, and B12; thiamin; riboflavin; magnesium; zinc; iron; copper; potassium; and iodine was lower than the recommended standard. Our own results regarding the successive decrease in oral food intake are consistent with those obtained by other authors [22,23,24]. In one study, on the day of radiotherapy initiation, 96.3% of the patients consumed more than 50% of the volume of meals administered, while on the day of the completion of radiotherapy, this percentage dropped to 38.9%. The patients who reported consuming less than 50% of their meal volume were characterized by a greater degree of malnutrition compared to the patients who took in more than 50% of their due portion of food [8].

In the available literature, there are few papers devoted to a detailed quantitative and qualitative analysis of the oral diet of head and neck cancer patients treated with radical radiotherapy. The present study not only attempted such an analysis, but also compared the effect of the addition of FSMP and enteral nutrition (as indicated) to the daily oral diet on the ability to meet daily energy requirements. The patients, due to the implementation of the recommendations at the beginning of therapy, took 585 kcal/d additionally to the oral diet, which translated into a total energy intake of 2432 kcal/d on average, while at the end of treatment, they took an additional 1080 kcal/d, resulting in a total average energy intake of 1890 kcal/d. With the inclusion of FSMP and enteral nutrition as indicated, the decrease in energy intake over the course of treatment was not as steep and drastic compared to the energy intake from the diet alone (a deficit of 543 kcal vs. 1038 kcal, respectively). Due to the fact that the patients received individually selected supplementation, they realized as much as 95.5% of their energy requirements in the last week of treatment, while from the oral diet alone the realization remained at only 40.9%. The inclusion of supplementation in the diet also improved the daily intake of protein (an additional 30 g of protein at the beginning of therapy and 56 g of protein at the end of therapy) and other macronutrients, as well as vitamins and minerals. Also of note in our study is the dynamic decline in the patients’ ability to eat orally. At the beginning of treatment, 94% of the patients were eating completely by the oral route; on the day of the end of therapy, only 49% were doing so. Due to aphagia, almost 30% of the patients on the day of the end of therapy were unable to take even a small amount of fluids by the oral route. This is in line with the results presented in the study by Citak et al. [8], in which 87% of patients were fed by the oral route at the beginning of therapy, and at the end of therapy this percentage dropped to 35.2% (statistically significant change).

According to our own research and a systematic review of the literature by Nugent et al. [25], relying solely on oral diets in the nutrition of head and neck cancer patients undergoing radical radiotherapy does not ensure that individual energy and protein requirements are met. The literature data confirm the positive effect of dietary intervention with special medical use foods on the nutritional status of patients treated with radiation therapy [8,25,26,27,28,29]. Ravasco et al. [30] showed that both dietary consultation and the use of FSMPs have a positive effect on ensuring the maintenance of adequate energy and protein intake in head and neck cancer patients treated with radical radiotherapy. In a study conducted by other authors, 13% of the patients were already on industrial diets at the start of therapy, and this percentage increased to 65% during therapy (FSMP -57.5%, industrial diet administered by nasogastric tube—7.5%). Patients who did not receive additional support using FSMP or enteral nutrition were statistically significantly more likely to be malnourished than those receiving FSMP or enteral nutrition [8]. Thus, the use of properly selected and well-managed supplementation with foods for special medical purposes and enteral nutrition with industrial diets may be helpful in improving the nutritional status of head and neck cancer patients treated with radical radiotherapy [25,26,27,28].

In order to improve the realization of daily energy requirements in patients with head and neck cancer, it seems reasonable to calculate individual energy requirements and fill the energy gap with FSMP and enteral nutrition, rather than following a general scheme, e.g., “two FSMPs for each patient for the entire treatment period”.

In a large proportion of head and neck cancer patients, despite the use of dietary counseling supported by FSMP at a certain stage of therapy, due to the severity of dysphagia, nutrition by oral route becomes insufficient or completely impossible and the patient requires the inclusion of enteral nutrition through artificial access. The criterion for choosing the type of enteral feeding access is the planned feeding time. If the planned period of enteral feeding is longer than 30 days, a PEG should be inserted according to established practice. When the planned duration of enteral feeding is less than 30 days, the access of choice is a nasogastric tube. The use of PEGs undoubtedly has more advantages over the use of tubes. These include the aforementioned possibility of longer, essentially indefinite enteral feeding, and as practice shows, patients often require enteral feeding for several more months after the end of radiotherapy. In addition, it is a more comfortable access for the patient, possible to hide under clothes, not stigmatizing the patient. By creating a PEG prior to treatment, it is possible to start feeding the patient at the exact moment when treatment toxicity begins to significantly result in reduced oral intake, without waiting for the access to be established and the location confirmed, as with the use of tubes [31,32]. Gavages, due to the risk of sores in the gastrointestinal tract, are removed approximately four weeks after insertion. Other complications associated with the use of gavages include changes in the nasal wing, chronic sinusitis, gastroesophageal reflux, and aspiration pneumonia. Furthermore, it is quite easy to have an untargeted gavage removed, and the time until the next probe is inserted is when no food can be administered, which is tantamount to starving the patient [31,32]. Prophylactic PEG insertion is practiced in head and neck cancer patients scheduled for treatment with radical radiation therapy, but it is not routine. At the National Institute of Oncology in Warsaw, where this study was conducted, according to established practice, most patients are offered prophylactic PEG insertion prior to radical radiotherapy. However, a significant number of patients refuse to have the access inserted. It should be noted that due to numerous complications, including death, a PEG is not inserted during treatment with radical radiotherapy. In a situation where a patient requires the initiation of enteral feeding during radical radiotherapy, and has not had a PEG created prior to treatment, the only option left is to insert a tube.

In the success of dietary intervention, a large role is played by the so-called compliance, i.e., the degree to which the patient adheres to the recommendations of medical personnel. In our study, those adhering to dietary recommendations (realizing at least 75% of the recommended dose of FSMP supplementation or enteral nutrition) had a statistically significantly lower mean weight loss compared to those not adhering to dietary recommendations (3.07 kg vs. 5.56 kg). The average compliance rate with dietary recommendations was 78%. In addition, there was a statistically significant negative association between weight loss during the course of radiation therapy and the degree of the implementation of recommendations. The increase in the implementation of recommendations was accompanied by a significant decrease in weight loss. The patients adhering to the recommended intake of the recommended amount of FSMP and enteral nutrition had less weight loss because they had a higher realization of daily energy, protein, and other macronutrient and micronutrient requirements compared to the patients not adhering to the recommendations. This is consistent with the results presented by Hubbard et al. [33], who conducted a meta-analysis of 46 studies and found that the clinical effect of taking FSMPs depends on the degree of patients’ compliance with the recommendations. The level of compliance with recommended FSMP supplementation in patients overall (inpatients and nursing home residents) was 78%, while it was 67% in hospitalized patients. The highest compliance with recommendations was observed in patients using liquid formulas, which are less filling and easier to ingest compared to other forms of administration, and high-energy formulas, which are lower in volume compared to isocaloric formulas. Clinical benefits were noted when energy intake from nutritional supplements was in the range of 250–600 kcal/d and the duration of use was at least 5 weeks. On the other hand, Hopanci et al. [27] reported that parameters such as BMI value, body weight, fat mass, and muscle mass significantly decreased during treatment in the group of patients who did not adhere to dietary recommendations and FSMP supplementation compared to the adherent group. In addition, severe oral mucositis was observed less frequently in the compliant patients (11.1%) compared to the non-compliant patients (88.9%).

The reasons for patients’ non-adherence to recommended FSMP supplementation and enteral nutrition remain under discussion. In our own study, the patients cited feelings of fullness (35.7%), nausea (33.3%), and taste disapproval (28.6%) as reasons for not adhering to the recommended supplementation. In contrast, in a study by Orell et al. [34], the most common included nausea (22% of the subjects), early satiety (12% of the subjects), loss of motivation (9% of the subjects), or others including diarrhea, financial problems, and weakness (21% of the subjects). Thus, it is extremely important to have a dietitian’s constant supervision over the amount of FSMPs taken and the implementation of enteral nutrition.

## 5. Limitations of the Study

In planning the methodology of the study, a prospective study using a control group was abandoned. In view of the knowledge regarding the impact of the negative effects of malnutrition on the patient’s general condition and the effectiveness of cancer therapy, it would have been unethical to isolate a group of patients who did not receive dietary counseling.

In the study, a historical control group was selected; however, the only parameter available from the area of nutritional status was body weight and information on whether there was any nutritional support (dietary consultation, FSMP, or enteral nutrition). The other parameters that were analyzed in the study group were not determined or information about them was incomplete in the analyzed medical records of patients in the historical control group.

## 6. Conclusions

Radical radiotherapy resulted in a systematic decrease in the average daily intake of energy and macro- and micronutrients in the following weeks of treatment.

The inclusion of FSMP and enteral nutrition in the diet significantly increased the percentage of meeting the patients’ energy requirements.

The fourth week of radiotherapy is the critical moment of treatment, when the greatest weight loss occurs along with the inability to consume 60% of the daily energy requirements.

The patients’ adherence to dietary recommendations (FSMP supplementation and enteral nutrition) was associated with decreased weight loss during radical radiotherapy.

Regular supervision by a dietitian (including the use of FSMP and enteral nutrition as indicated) of head and neck cancer patients undergoing radical radiotherapy should be routine during anticancer treatment.

## Figures and Tables

**Table 1:** Basic characteristics of the study group.

Variable	Feature	N	%	*p* *
Sex	man	78	75.0	<0.001
	woman	26	25.0	
Age (years)	20–29	3	2.9	<0.001
	30–39	6	5.8	
	40–49	10	9.3	
	50–59	26	25.0	
	60–69	47	45.2	
	70–79	12	11.5	
	80+	0	0	
Body weight (kg)	<45	0	0	<0.001
	45–54	5	4.8	
	55–64	22	21.2	
	65–74	31	29.8	
	75–84	15	14.4	
	85–94	19	18.3	
	≥95	12	11.5	
BMI (kg/m)^2^	<18.5	2	1.9	<0.001
	18.5–24.9	48	46.2	
	25–29.9	35	33.7	
	≥30	19	18.3	
Operation	yes	39	37.5	0.011
	not	65	62.5	
Induction chemotherapy	yes	34	32.7	<0.001
	not	70	67.3	
Type of therapy	rth	39	37.5	<0.001
	rth-chth	61	58.7	
	rth-Cetuximab	4	3.8	
The manufacture of a PEG before treatment	yes	29	27.9	<0.001
	not	75	72.1	
Alternative supplementation or diet without medical indication	yes	5	4.8	<0.001
	not	99	95.2	

rth—radiotherapy; rth-chth—radiochemotherapy; rth-Cetuximab—radiotherapy with Cetuximab; PEG—percutaneous endoscopic gastrostomy; * χ² test.

**Table 2:** Average intake of energy and macro- and micronutrients of the diet without FSMP.

Component	Unit	T2	T3	T4	T5	T6	T6 as % of T2	*p*
Energy	kcal	1847.01	1467.76	1221.29	993.33	809.47	43.83%	<0.001
Total protein	g	70.31	60.87	53.73	44.51	36.55	51.99%	<0.001
Animal protein	g	38.00	35.21	33.05	27.00	22.54	59.32%	<0.001
Plant protein	g	31.52	24.94	19.97	16.97	13.46	42.69%	<0.001
Grease	g	49.98	39.61	34.80	26.89	22.54	45.09%	<0.001
Total carbohydrates	g	290.05	226.32	180.77	149.63	120.23	41.45%	<0.001
Sodium	mg	4060.48	3617.95	2957.83	2576.32	1996.97	49.18%	<0.001
Potassium	mg	2791.74	2442.03	2160.15	1822.65	1476.05	52.87%	<0.001
Calcium	mg	504.32	526.58	591.74	483.52	393.49	78.02%	0.001
Phosphorus	mg	1181.67	1043.87	987.71	819.72	674.41	57.07%	0.001
Magnesium	mg	312.48	268.66	236.14	206.18	162.11	51.88%	<0.001
Iron	mg	10.40	8.67	7.15	6.06	4.89	47.05%	<0.001
Zinc	mg	9.15	7.79	6.55	5.58	4.56	49.81%	<0.001
Copper	mg	1.16	0.94	0.81	0.67	0.53	45.73%	<0.001
Manganese	mg	3.78	3.36	2.98	2.48	2.05	54.18%	<0.001
Vitamin A (retinol eq.)	µg	1622.90	1299.49	1116.05	855.76	679.38	41.86%	<0.001
Vitamin E (alpha-tocopherol eq.)	mg	5.08	4.20	3.77	2.75	2.29	45.07%	<0.001
Thiamine	mg	1.22	0.94	0.82	0.69	0.55	44.94%	<0.001
Riboflavin	mg	1.41	1.26	1.28	1.02	0.84	59.72%	<0.001
Niacin	mg	15.34	11.09	9.35	7.75	6.16	40.15%	<0.001
Vitamin B6	mg	1.83	1.52	1.29	1.09	0.88	48.04%	<0.001
Vitamin C	mg	50.94	44.03	36.22	29.74	22.47	44.12%	<0.001
Fatty acids: total saturated	g	24.64	19.28	16.45	13.46	11.24	45.63%	<0.001
Fatty acids: total monounsaturated.	g	15.05	12.39	11.00	8.09	6.92	45.96%	<0.001
Fatty acids: total polyuns.	g	6.29	4.80	4.69	3.28	2.60	41.31%	<0.001
Cholesterol	mg	284.49	211.80	169.15	137.37	126.64	44.51%	<0.001
Dietary fiber	g	22.06	18.78	14.84	12.53	10.20	46.24%	<0.001
Folates	µg	228.21	197.60	170.96	146.28	114.73	50.27%	<0.001
Vitamin B12	µg	3.43	2.57	2.62	2.18	1.81	52.75%	<0.001
Vitamin D	µg	2.53	1.29	1.21	0.86	0.81	32.24%	<0.001
Iodine	µg	169.84	162.06	141.98	129.09	95.40	56.17%	<0.001
Long-chain polyunsaturated fatty acids	g	0.44	0.14	0.14	0.12	0.10	23.39%	<0.001
Omega-6 fatty acids	g	4.54	3.78	3.51	2.47	2.07	45.60%	<0.001
Omega-3 fatty acids	g	1.75	1.01	1.18	0.81	0.53	30.14%	<0.001
Percentage of energy from protein	%	15.82	16.93	14.15	15.83	14.04	88.77%	n/a
Percentage of energy from fat	%	23.79	23.21	24.15	20.84	18.22	76.60%	n/a
Percentage of energy from carbohydrates	%	57.13	54.54	52.23	45.50	38.27	66.99%	n/a

T2—second week of the study; T3—third week of the study; T4—fourth week of the study; T5—fifth week of the study; T6—sixth week of the study; n/a—not applicable.

**Table 3:** Average intake of energy and macro- and micronutrients of the diet including FSMP and enteral nutrition.

Component	Unit	T2	T3	T4	T5	T6	T6 as % of T2	*p*
Energy	kcal	2432.21	2111.24	1943.32	1860.67	1889.87	77.7%	<0.001
Total protein	g	100.00	92.75	89.51	87.82	92.43	92.4%	0.002
Animal protein	g	38.00	35.21	33.05	27.00	22.54	59.3%	<0.001
Plant protein	g	31.52	24.94	19.97	16.97	13.46	42.7%	<0.001
Grease	g	79.48	73.04	73.54	74.26	78.98	99.4%	0.149
Total carbohydrates	g	339.62	279.22	237.25	214.99	205.18	60.4%	<0.001
Sodium	mg	4174.30	3751.54	3119.55	2786.50	2341.84	56.1%	<0.001
Potassium	mg	3068.63	2761.17	2544.38	2322.49	2238.41	72.9%	<0.001
Calcium	mg	1196.88	1260.67	1397.85	1438.53	1589.70	132.8%	<0.001
Phosphorus	mg	1747.47	1633.09	1607.98	1521.39	1518.64	86.9%	<0.001
Magnesium	mg	419.35	381.13	358.11	347.27	342.61	81.7%	<0.001
Iron	mg	15.36	14.23	14.03	15.04	17.86	116.3%	0.051
Zinc	mg	14.34	13.46	13.12	13.75	15.69	109.4%	0.008
Copper	mg	172.83	253.71	517.46	866.66	1484.95	859.2%	0.214
Manganese	mg	5.11	4.81	4.67	4.56	4.89	95.7%	0.213
Vitamin A (retinol equiv.)	µg	2173.28	1896.09	1805.08	1710.40	1809.62	83.3%	0.002
Vit. E (alpha-tocopherol equiv.)	mg	13.62	13.67	14.82	16.74	21.20	155.7%	0.085
Thiamine	mg	2.16	1.94	1.94	2.03	2.28	105.9%	0.012
Riboflavin	mg	2.45	2.38	2.59	2.63	2.98	121.3%	0.416
Niacin	mg	22.28	18.37	17.17	16.68	17.66	79.3%	<0.001
Vitamin B6	mg	2.97	2.76	2.70	2.83	3.18	107.1%	0.073
Vitamin C	mg	112.24	109.57	108.83	116.31	134.11	119.5%	0.210
Fatty acids: total saturated	g	28.72	24.30	23.79	24.05	26.53	92.4%	0.003
Fatty acids: total monounsaturated.	g	15.83	13.57	13.45	12.13	14.69	92.8%	0.004
Fatty acids: total polyuns.	g	7.15	6.05	7.25	7.58	9.86	137.9%	0.016
Cholesterol	mg	284.49	211.80	169.15	137.37	126.64	44.5%	<0.001
Dietary fiber	g	22.65	19.56	15.31	12.91	10.57	46.7%	<0.001
Folates	µg	228.21	197.60	170.96	146.28	114.73	50.3%	<0.001
Vitamin B12	µg	5.65	4.93	5.28	5.39	5.88	104.2%	0.144
Vitamin D	µg	3.99	3.37	4.66	6.42	9.48	237.3%	<0.001
Iodine	µg	265.43	265.15	262.00	278.16	291.72	109.9%	0.363
Long-chain polyunsaturated fatty acids	g	0.44	0.14	0.14	0.12	0.10	23.4%	<0.001
Omega-6 fatty acids	g	4.54	3.78	3.51	2.47	2.07	45.6%	<0.001
Omega-3 fatty acids	g	1.75	1.01	1.18	0.81	0.53	30.1%	<0.001

T2—second week of the study; T3—third week of the study; T4—fourth week of the study; T5—fifth week of the study; T6—sixth week of the study.

**Table 4:** Comparison of average daily energy intake (kcal) to energy requirements in consecutive weeks of treatment.

		T2	T3	T4	T5	T6
Energy requirement	kcal	2019.1	2009.0	1998.9	1993.0	1978.1
Consumption without FSMP and EN	kcal	1847.0	1467.8	1221.3	993.3	809.5
	% of demand	91.5	73.1	61.1	49.8	40.9
	*p*	0.023	<0.001	<0.001	<0.001	<0.001
Consumption with FSMP and EN	kcal	2432.2	2111.2	1943.3	1860.7	1889.9
	% of demand	120.5	105.1	97.2	93.4	95.5
	*p*	<0.001	0.107	0.628	0.171	0.408

T2—second week of the study; T3—third week of the study; T4—fourth week of the study; T5—fifth week of the study; T6—sixth week of the study.

**Table 5:** Share of people consuming by oral route more than 60% of daily energy requirements, not including FSMP and enteral feeding.

	T1	T2	T3	T4	T5	T6
N	104	104	104	104	104	104
N yes	98	88	68	49	42	31
% Yes	94.2	84.2	66.0	47.4	40.6	29.7
N no	6	16	36	55	62	73
% No	5.8	15.8	34.0	52.6	59.4	70.3

T1—first week of study; T2—second week of study; T3—third week of study; T4—fourth week of study; T5—fifth week of study; T6—sixth week of study.

**Table 6:** Daily energy values of recommended and actual FSMP intake and EN (kcal), and the difference between these values in subsequent weeks of treatment (kcal) (n = 104).

T	M/SD	Energy Value Recommended FSMP/EN [kcal]	Energy Value Taken FSMP/EN [kcal]	Difference between the Energy Value of the Recommended and Taken FSMP/EN [kcal]	*p*
T2	M	749.7	595.8	153.0	<0.001
	SD	332.70	420.34	291.59	
T3	M	793.8	662.4	137.7	<0.001
	SD	343.76	424.08	273.69	
T4	M	947.8	733.80	214.0	<0.001
	SD	416.46	481.24	317.44	
T5	M	1141.5	893.2	248.4	<0.001
	SD	511.94	541.19	357.15	
T6	M	1340.4	1096.8	243.7	<0.001
	SD	545.2	579.47	465.60	

FSMP—food for special medical purposes; EN—enteral nutrition; T—study week; T2—second study week; T3—third study week; T4—fourth study week; T5—fifth study week; T6—sixth study week.

**Table 7:** Weight loss (kg) from T1 to T6 in patients according to adherence to dietary recommendations.

	N	M	Me	SD	*p*
Total	104	4.03	4.00	3.03	
Compliant	64	3.07	3.00	2.32	*p* < 0.001
Non-compliant	40	5.56	5.25	3.40	

## Data Availability

The data presented in this study are available on request from the corresponding author. The data are not publicly available due to privacy reasons.
